# Cutting-edge greenhouse practices for better stigma yield and corm quality of saffron (*Crocus sativus* L.)

**DOI:** 10.3389/fpls.2025.1612791

**Published:** 2025-06-24

**Authors:** Muien Qaryouti, Abdelrahman Al-Soqeer, Mohamed E. Abdelaziz, Nazim S. Gruda, Saif AlSahly, Wafa Alrasheed, Sahar Althobiti, Omar Babiker, Mahmoud Sharafeldin, Wim Voogt

**Affiliations:** ^1^ National Research and Development Center for Sustainable Agriculture (Estidamah), Riyadh, Saudi Arabia; ^2^ Faculty of Agriculture, Cairo University, Giza, Egypt; ^3^ Division of Horticultural Sciences, Institute of Crop Science and Resource Conservation, University of Bonn, Bonn, Germany; ^4^ Department of Agricultural Research and Extension, ‏Ministry of Environment & Water and Agriculture, Riyadh, Saudi Arabia; ^5^ Non-Traditional Spices Biotechnology Unit, Department of Medicinal and Aromatic Plants Research, National Research Centre, Cairo, Egypt; ^6^ Business Unit Greenhouse Horticulture, Wageningen University and Research, Wageningen, Netherlands

**Keywords:** saffron (C. sativus), planting depth, planting density, stigma yield, new corms

## Abstract

Saffron (*Crocus sativus* L.) is among the world’s most expensive spices, prized for its red stigmas used as a flavoring and a natural dye. Saudi Arabia is a significant importer of saffron, but the high cost of importing quality corms makes it economically unfeasible relative to the potential income from saffron production. Additionally, the high temperatures and harsh conditions of open fields pose significant challenges for saffron cultivation in the region. We investigated saffron cultivation under controlled greenhouse conditions with cooling to address these issues. Our study examined three plant densities—200, 100, and 67 corms m^-^²—and two planting depths—8 cm and 13 cm—to assess their effects on plant growth, flower yield, stigma production, and new corm development. We found that higher plant density (200 corms m^-^²) increased flower, and stigma yields per unit area but decreased flower number, stigma production, and plant weight per individual plant. Deeper planting (13 cm) reduced new corm production, particularly at the highest density. The largest corms and the highest percentage of big corms were observed at the lowest density (67 corms m^-^²), with planting depth having minimal impact on corm production. Given the high cost of quality corms, balancing flower production per corm with reproductive capacity is crucial. Therefore, based on our findings, we recommend a moderate planting density of 100 corms m^-^² and a shallow planting depth of 8 cm. These conditions provide a more balanced approach, optimizing both flower yield and corm production. Implementing these recommendations could enhance the efficiency and sustainability of saffron cultivation in greenhouses with cooling, making it a viable option for regions with challenging growing conditions.

## 1 Introduction

Saffron (*Crocus sativus* L.) is one of the highest-priced spices in the world (Winterhalter and Straubinger, 2000). It is grown for its red scarlet stigmas, which are used as a spice and natural dye. The price of dry saffron stigma varies from 1,500 to 2,200 Euro/kg (Mykhailenko et al., 2020). Saffron has been successfully grown in different geographic locations throughout the world. This crop can be cultivated in temperate, semi-arid, and arid climatic conditions (Kumar et al., 2009). However, in traditional cultivation, variations in average dry stigma yield between countries were recorded, ranged from 2.0 kg ha^-1^ in Morocco and Spain to 2.29 kg ha^-1^ in India, 4 kg ha^-1^ in Iran, and 4.3 kg ha^-1^ in Greece, while the highest yield, 8.18 kg ha^-1^, was recorded in Italy (Menia et al., 2018). This variation in productivity and quality is likely due to growing conditions and farmers’ cultural practices (De Juan et al., 2009; Asil and Ayanoglu, 2018; Sevindik et al., 2020; Maggi et al., 2011). Temperature, photoperiod, topographical locations, altitude (Siracusa et al., 2010; Rahimi et al., 2017; Kafi and Showket, 2007), and soil properties (Cardone et al., 2020) are the critical environmental conditions that affect saffron production.

Menia et al. (2018) reported that saffron flower number per unit area depends on many factors, including climatic conditions like temperature regime and rainfall, soil conditions, quality of planting material, and agricultural practices. Gresta et al. (2009) stated that, saffron flower initiation is affected by the interaction of air temperature and soil moisture, and they concluded that colder environments lead to higher flower production due the effect of low temperature on flower initiation and less water stress, however, the timing of flowering is independent from planting density. Bayat et al. (2016) reported that climatic conditions are one of the most important factors determining the yield of saffron, which limits the cultivation in different parts of the world. Pirasteh-Anosheh et al. (2023) reported that lower temperatures, especially before flowering, positively affected yield. Low air temperature accelerates dormancy interruption in the mother corms for a new growing season (Rashed-Mohassel, 2020), inducing central corm buds and the appearance of root primordia at the base of corms (Negbi, 2006). Higher temperatures postpone flowering (Rashed-Mohassel, 2020), decrease flower number, and reduce yield (Koocheki and Khajeh-Hosseini, 2020; Negbi, 2006; Pirasteh-Anosheh et al., 2022). Pirasteh-Anosheh et al. (2023), reported that autumn temperature is essential for stigma yield and spring temperature for determining new corms yield for the following year.

Besides environmental conditions, saffron yield could be further improved by optimization of agronomic factors such as production system, corm size, planting depth, and planting density (De Juan et al., 2009; Andabjadid et al., 2015). Andabjadid et al. (2015) and Nazarian et al. (2016) reported that saffron yield substantially depends on corm density and size. Under plastic tunnel conditions, Mollafilabi et al. (2013) reported that the highest yield of 7.38 kg ha^-1^ saffron dried stigma was obtained from soil cultivation and planting density of 150 corms m^-2^, compared with planting densities of 50 and 100 corms m^-2^. Increasing plant density from 60 to 150 corms m^-2^ significantly increased the number of flowers and stigma dry weight per m^-2^ by 174 to 192 percent in the first year, 100 to 109 percent in the second year, and 128 to 129 percent in the third year, respectively (Sharifi et al., 2021). Gheshm and Brown (2021) reported that increasing planting density from 120 to 162 corms m^-2^ had no significant effect on flower number, stigma yield, or pistil dry weight. The impact of saffron planting depth on flowering, stigma yield, and formation of new corms varied with environmental conditions (Gheshm and Brown, 2021) and crop cycle (Yildirim et al., 2017). Shallow planted corms of 5 cm depth proportionally increased the formation of new corms but had no significant effect on stigma yield in the first year, while in the second-year planting at 15 cm depth, they markedly increased new corm circumference, improved the number of flowers, and stigma yield (Yildirim et al., 2017). Under climatic conditions where snow cover is long-lasting, planting corms at 10 to 15 cm depth between late July and the end of August accelerated shoot and flower emergence (Ayari et al., 2022). Farmers in central Italy recommended planting corms at medium density (111 to 119 corms m^-2^) due to the similar stigma yield and the highest production of daughter corms produced compared to high density (139 to 179 corms m^-2^ (Temperini et al., 2009). These authors also stated that, in a Mediterranean environment, appropriate crop techniques, e.g., lifespan and plant density), can improve the quantitative characteristics of saffron.

Saudi Arabia is one of the largest saffron importing countries. In 2020, about 125 tons of dry stigma were imported (The General Authority for Statistics, 2020), and this demand is expected to increase in the coming years. In recent years, local farmers have tried introducing saffron cultivation in different regions in Saudi Arabia (SA). Saffron cultivation in Saudi Arabia faces significant limitations due to extreme environmental conditions, and traditional open field methods are not viable. Average day temperatures in this region ranged from 20 to 35°C during September to December and from 10 to 32 °C during January to Apr. (WB CCKP, 2021). Furthermore, the high cost of imported corms obstructs the economic feasibility of Saudi saffron production. Therefore, saffron cultivation under controlled greenhouse conditions with full temperature and humidity control by cooling, screening, and humidification provides good potential for controlled saffron production, aiming at high yields and quality of stigma, and might be an alternative for the application of saffron cultivation in such regions.

Furthermore, despite the many studies cited above on the effect of planting density and depth in other climates, they do not reveal how these factors influence saffron growth and development and the subsequent yield of flowers, stigma, and corms under controlled greenhouse conditions in desert regions. Therefore, this study aims to investigate the effect of planting densities and planting depth on saffron growth, stigma yield, and new corm development under controlled greenhouse conditions in the hot and arid climate of Saudi Arabia.

## 2 Materials and methods

### 2.1 Greenhouse and experimental site

**Figure 1:**
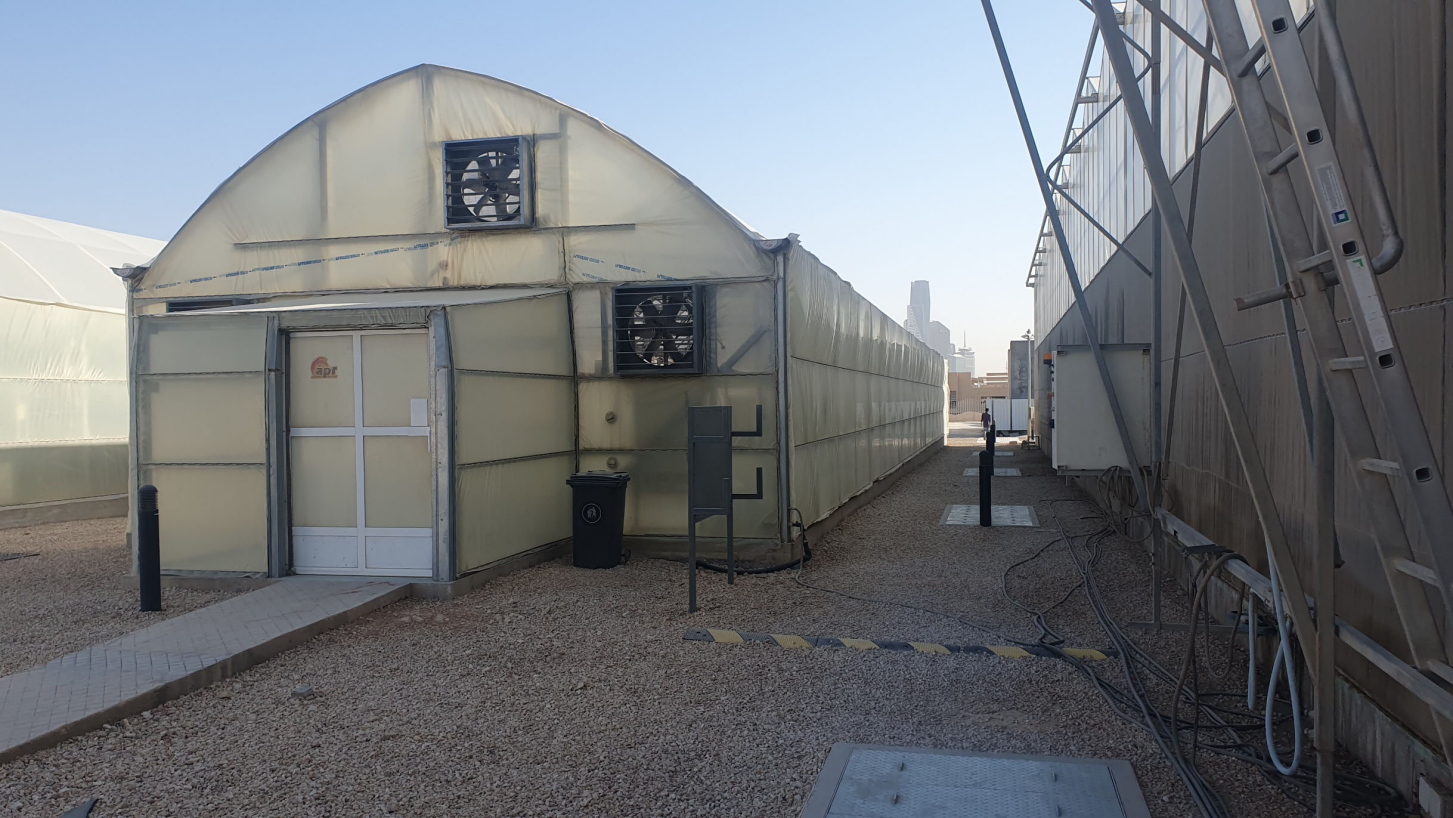
View of the greenhouse where the described experiment took place.

**Figure 2:**
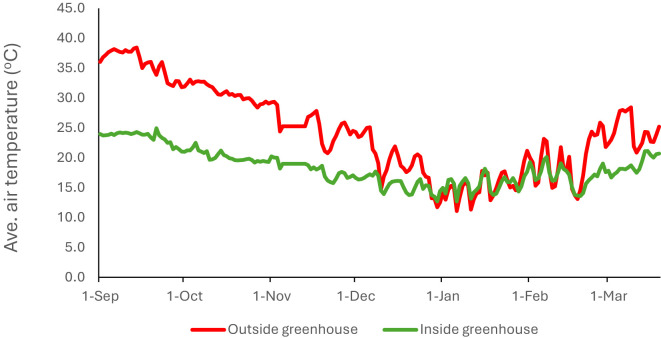
Average air temperature inside and outside the greenhouse during the growing season of saffron.

In this experiment, we used one greenhouse compartment at the National Research and Development Center for Sustainable Agriculture (ESTIDAMAH), Riyadh, Saudi Arabia (46°37’ E longitude and 24°39’ N latitude). A single plastic tunnel greenhouse (**Figure 1**), measuring 40 m × 8 m with a growing area of 288 m², had a gutter height of 3 m and a total height of 5.4 m. The structure was covered with polyethylene film and equipped with a pad and fan cooling system. This system included a cellulose pad wall (8 m × 2 m × 15 cm) and three fans, each with a 15,000 m³ h^-^¹ capacity, with two fans placed 2 m above the ground, and the third at 3.5 m (**Figure 1**). Together, they provided a total air exchange rate of 140.6 m³ m^-^² h^-^¹, enabling up to 33 complete air exchanges per hour. The fan speed was controlled, independently and automatically by a greenhouse process control computer (Ridder/HortiMaX MultiMa), which controlled the climate and provided data collection by using three ventilated temperature and humidity sensors Greenhouse temperature was maintained between 20 and 25°C during the day and between 18 and 20°C at night (**Figure 2**). The relative air humidity in the greenhouse remained around 70–80% during the day, depending on the operation of the pad and fan system.

### 2.2 Plant material

As a certified source for saffron corms is unavailable locally, corms were imported from Bloembollenbedrijf. J.C. Koot, Netherlands, with an average weight of 25 g corm^-1^. The planting area was prepared manually, with three raised beds measuring 30 m^2^ (30x1m). Corms were planted on September 17, 2022, at three densities: high density (200 corms m^-^²), moderate density (100 corms m^-^²), and low density (67 corms m^-^²), and at two depths—8 cm and 13 cm from the corm bottom.

### 2.3 Experimental design

**Figure 3:**
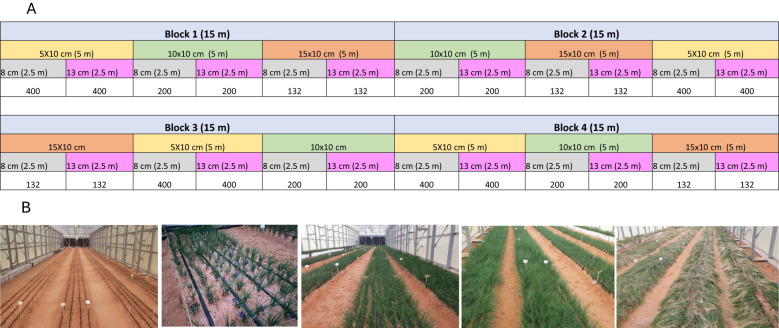
Experiment layout and saffron growth stages in the greenhouse where the experiment took place.

A split-plot experimental design with four replicates was used for each 2.5 m² experimental unit. Plant densities were assigned for the main plots, and planting depth was assigned for the sub-plots (**Figure 3**).

### 2.4 Irrigation and fertilizer management:

The fertigation solution was supplied through drip irrigation, (Netafim KAMELEON, 2l/hr.) with 2.5 drippers m^-2^, which was controlled by the Hortimax computer. Fertigation amount was based on the cumulative radiation sum per day and was initiated when global radiation sum exceeded 800 J cm^-^² and adjusted with one shot (80 ml) per dripper for each 100 J cm^-2^ increase in radiation. The fertilizer recipe was based on the nutrient solution in **Table 1** and composed from commercially available fertilizers, following the methodology outlined by Sonneveld and Voogt (2009). The fertilizer stock solution was prepared at a 150-times concentrated level. Iron (Fe) was applied in the form of EDDHA chelate, while manganese (Mn) and zinc (Zn) were applied as DTPA chelates. For macronutrients, straight fertilizer salts were used, including calcium nitrate, potassium nitrate, monopotassium phosphate, monoammonium phosphate, and magnesium sulphate. Tap water, demineralized by reverse osmosis (EC < 0.1 mS cm^-^¹), was used as the water source. An automatic dosing system then diluted the stock solution to a target EC of 1.5 and regulated pH at 5.8.

**Table 1:** Nutrient solution used for the saffron trials, with a reference EC of 1.5 mS/cm.

*mmol/l*									*µmol/I*					
NH4	K	Ca	Mg	Si	NO3	Cl	SO4	P	Fe	Mn	Zn	B	Cu	Mo
0.5	6.9	2.5	1.1	0.0	10.8	0.1	1.4	1.1	25	15	4.0	25	0.9	0.4

The pH and EC of the irrigation and the concentration of each macro and micronutrient were monitored regularly to ensure that the crop would not suffer from a deficiency.

### 2.5 Data collection:

Saffron flowers were harvested manually every 1-2 days from each experimental unit/treatment during the flowering period (7^th^ to 28^th^ November 2022). The number of harvested flowers was recorded. Afterward, flowers were taken to the laboratory, where stigmas were manually separated from flowers. Stigmas were dried at room temperature (21-25°C) for 48 hours to determine the dry yield using an analytical digital balance (METTLER-TOLEDO GmbH, Model: XSR205DU). On December 3rd, ten random plants from each replicate in each treatment were harvested to determine the number and fresh weight of leaves and corms using a digital balance (CAMRY Model: ACS-3_ZE20).

## 3 Results

### 3.1 Greenhouse climate

The daily average greenhouse temperature was consistently lower than the outside temperature throughout the saffron growing period (**Figure 2**). In particularly during the first two months (Sept – Oct) where the temperature inside the greenhouse was up to 15 degrees lower.

### 3.2 Number of flowers and dry stigma yield

The results showed significant effect of plant density on average flowers number and dry stigma yield per m^2^. Flower number increased significantly from 340 and 337 flowers m^2^ to 553 and 600 flowers m^2^ and dry stigma yield from 3180 and 3161 mg to 5174 and 5614 mg m^2^ with increasing plant density from 67 to 200 plants m^2^ using 8 and 13 cm planting depth, respectively (**Table 2**). No significant differences in flower number and stigma yield were observed between the two planting densities 100 and 200 plants m^2^ in both planting depth (**Table 2**). Furthermore, planting depth has no significant effect on the total flower number and stigma yield m^2^.

**Table 2:** Effect of planting density and planting depth on average flower number and dry stigma yield plant^-1^ of saffron plants grown in soil under greenhouse conditions, 2022.

Planting depth (cm)	Planting density (plant m^2^)			Average
	67	100	200	
	No. of flower m^-1^			
8	339.8 ± 35.8 b*	501.4 ± 45.9 ab	553.0 ± 95.4 a	464.6 ± 45.5 a
13	337.3 ± 29.9 b	435.2 ± 31.0 ab	599.8 ± 78.2 a	457.8 ± 39.7 a
Average	338.1 ± 21.3 b	468.9 ± 33.4 ab	576.3 ± 57.8 a	
	Dry stigma yield (mg m ^1^)			
8	3180.2 ± 321.7 b	4696.5 ± 435.5 ab	5173.5 ± 927.4 a	4350.1 ± 427.3 a
13	3161.2 ± 275.7 b	4078.5 ± 301.9 ab	5613.8 ± 754.3 a	4284.5 ± 387.0 a
Average	3170.8 ± 172.3 b	4387.5 ± 266.9 ab	5393.6 ± 537.2 a	

*Values having different letters are signiﬁcantly different at 5% probability level.

**Figure 4:**
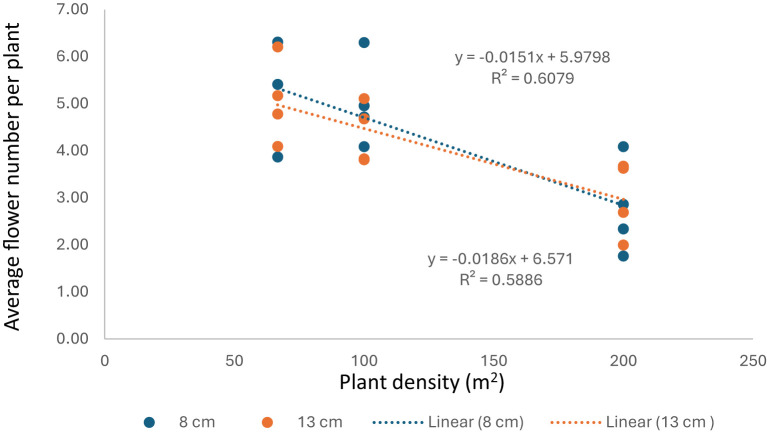
Relationship between plant density and flower number per plant^-1^.

Although, increasing plant density increased total flower number and dry stigma yield per m^2^, the results showed negative correlation between planting density and number of flowers plant^-1^ in the two-planting depth, indicating that, under high plant density, the plants are unable to produce same number of flowers and thereafter stigmas yield as the case under low planting densities (**Figure 4**).

### 3.2 Plant fresh weight and number of spathe per plant^-1^

The highest average plant fresh weight was produced from plants grown in low plant density (67 plants m^2^), with no significant differences with moderate plant density (100 plants m^2^), while the lowest significant plant fresh weight was produced when using high plant density (200 plants m^2^) and 8 cm planting depth (**Table 3**). The highest average spathe number per plant was produced from plants grown under moderate plant density, with no significant differences from those plants grown under low plant density (**Table 3**).

**Table 3:** Effect of planting density and depth on average plant fresh weight and spathe number of saffron plants grown in soil under greenhouse conditions, 2022.

Planting depth (cm)	Planting density (plant m^2^)			Average
	67	100	200	
	Plant fresh weight (g plant^-1^)			
8	45.0± 2.11ab*	42.4± 2.11 ab	31.4± 1.75 c	39.6± 2.03 a
13	46.3± 2.48 a	43.1 ± 3.26 ab	37.5 ± 1.94 b	42.3± 1.84 a
Average	45.7± 1.45 a	42.7± 2.06 a	34.5 ± 1.65 b	
	Ave. no. of spathe plant ^1^			Average
8	7.9 ± 0.30 ab	7.9± 0.47 ab	6.3± 0.30 c	7.9± 0.45 a
13	8.2± 0.47 ab	9.6± 0.66 a	8.7± 0.71 ab	8.3± 0.31 a
Average	8.1± 0.25 ab	8.8± 0.46 a	7.5± 0.55 b	

*Values having different letters are signiﬁcantly different at 5% probability level.

**Figure 5:**
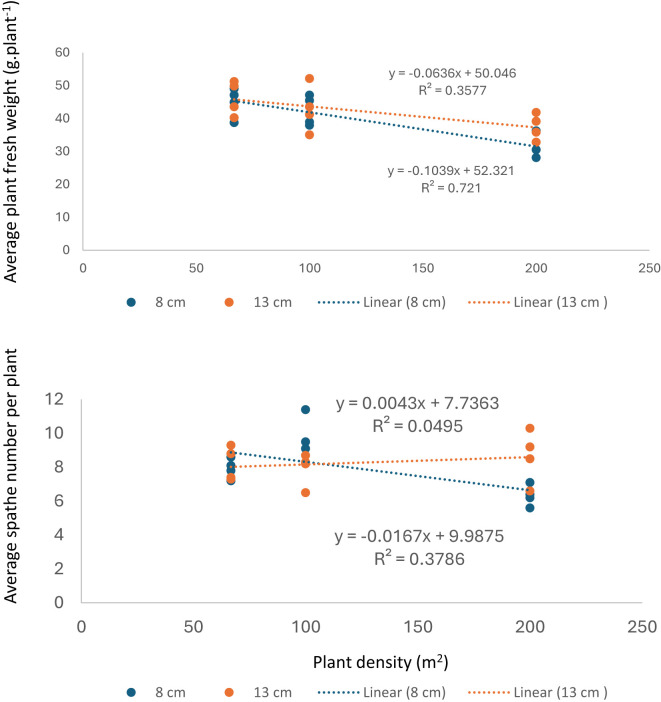
Relationship between plant density with plant fresh weight and spathe number plant^-1^.

There was a negative correlation between plant fresh weight and increasing planting density, and this correlation decreased with increasing planting depth. Furthermore, average spathe number per plant was less affected by increasing planting densities when using 8 cm planting depth, and there was no correlation between planting density and spathe number when using 13 cm planting depth (**Figure 5**).

**Figure 6:**
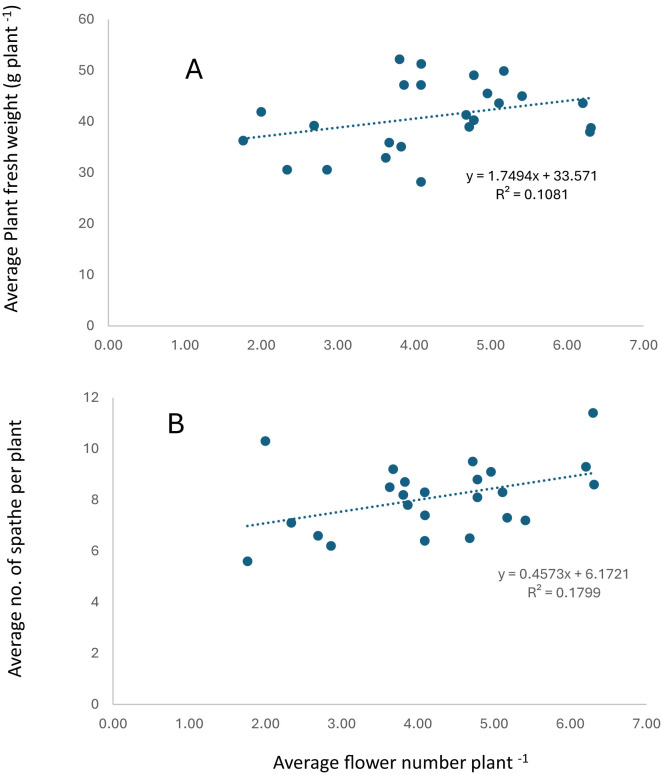
Relationship between plant fresh weight **(A)**, number of spathes **(B)** with flower number per plant.

Furthermore, the results showed a weak positive correlation between plant fresh weight, number of spathe plant ^1^ with average flower number plant^-1^ (**Figures 6A, B**).

### 3.3 New corms yield

New baby corms were harvested on Mar. 17, 2023, from each treatment and separated into three corm fractions based on corm weight: big size (above 5 g corm^-1^), medium size (between 3-5 g corm^-1^), and small size (below 3 g corm^-1^). Our results showed a significant effect of plant density on total new corms yield m^2^. The highest corm yield was produced from high plant density treatment, with no significant effect of planting depth and those plants grown in moderate plant density and 8 cm planting depth. The lowest corms yield was produced from low plant density (67 plants m^2^) with no significant differences between planting depth (**Table 4**).

**Table 4:** Effect of plant density and planting depth on average corms yield m^2^ saffron plants grown in soil under greenhouse conditions, 2023.

Planting depth (cm)	Planting density (plant m^2^)			Average
	67	100	200	
	Average new corms yield (kg m^2^)			
8	1.9± 0.16 d*	2.2± 0.19 abc	2.5 ± 0.14 a	2.2± 0.11 a
13	2.0 ± 0.08 cd	2.1± 0.20 bcd	2.4± 0.18 ab	2.2± 0.08 a
Average	2.0± 0.08 c	2.2± 0.12 b	2.4± 0.11 a	
	Average new corms number m^2^			Average
8	488.0± 14.2 c	590.5± 25.5 c	1012.5 ± 70.7 a	727.1± 74.6 a
13	479.5± 19.5 c	578.6± 69.7 c	846.3± 89.9 b	645.6± 63.3 a
Average	483.7± 11.0 c	584.5± 32.4 b	929.4± 63.1 a	

*Values having different letters are signiﬁcantly different at 5% probability level.

The total number of new corms m^2^ was also significantly affected by plant density (**Table 4**). The highest new corms was produced from high plant density and 8 cm planting depth. However, with increasing planting depth to 13 cm, the number of corms was reduced significantly. There were no significant differences in the number of corms between moderate and low plant densities in both planting depths (**Table 4**).

The highest percentage (32.3% and 28.8%) of big corms and the lowest percentage (36.6% and 32.1%) of small corms were produced by using low plant density and 13 cm or 8 cm planting depth, respectively (**Table 5**), whereas the lowest percentage of big new corms (17.8% and 19.1%) and the highest percentage of small corms (57.1% and 55.9%) were produced from high plant density.

**Table 5:** Effect of plant density and planting depth on the size of new corms.

Planting depth (cm)	Planting density (plant m^2^)							
	67		100		200		Average	
Average % of new big size corms (above 5 g per corm)								
	% Wt	% No.	% Wt	% No.	% Wt	% No.	Wt	No.
8	24.8± 1.3 b	21.8± 3.4 a	24.9± 1.9 b	20.4± 3.1 a	18.8± 3.6 c	7.2± 1.1 b	23.3± 1.5 a	17.1± 2.4 a
13	34.7± 4.0 a	21.9± 1.3 a	26.1± 4.6 b	22.1± 4.4 a	19.1± 4.7 c	9.4 ± 2.5 b	28.5± 2.7 a	18.3± 2.2 a
Average	29.8± 2.6 a	21.1 ± 1.7 a	25.5± 2.2 b	21.3 ± 2.3 a	18.5 ± 2.7 c	8.3 ± 1.4 b		
Average % of new medium size corms (3-5 g per corm)								
	67		100		200		Average	
8	28.0 ± 2.8 ab	34.6 ± 4.2 a	25.8 ± 2.2 b	30.8 ± 3.4 a	25.7 ± 5.5b	15.6 ± 1.6 b	26.8 ± 2.0 b	28.0 ± 2.9 a
13	35.6 ± 3.7 a	35.2 ± 2.4 a	27.8 ± 3.1 ab	35.6 ± 2.0 a	22.6 ± 3.1b	17.4 ± 2.0 b	30.2 ± 2.2 a	30.9 ± 2.4 a
Average	31.7 ± 2.5 a	34.9 ± 2.1 a	26.9 ± 1.8 ab	33.2 ± 2.0 a	24.8 ± 2.9b	16.5 ± 1.2 b		
Average % of new small size corms (less than 3 g per corm)								
	67		100		200		Average	
8	36.4 ± 4.9 b	55.8 ± 4.9 b	39.1 ± 5.8 b	54.7 ± 4.9 b	57.1 ± 9.1 a	72.5 ± 4.7 a	42.7 ± 3.9 a	61 ± 3.5 a
13	33.1 ± 6.1 b	48.1 ± 8.2 b	37.4 ± 5.7 b	59.9 ± 5.0 ab	55.9 ± 7.8 a	70.6 ± 6.6 a	44.3 ± 4.7 a	60 ± 4.0 a
Average	34.9 ± 3.7 b	51.9 ± 4.5 b	38.8 ± 3.5 b	57.3 ± 3.2 ab	56.9 ± 5.9 a	71.5 ± 3.8a		

**Figure 7:**
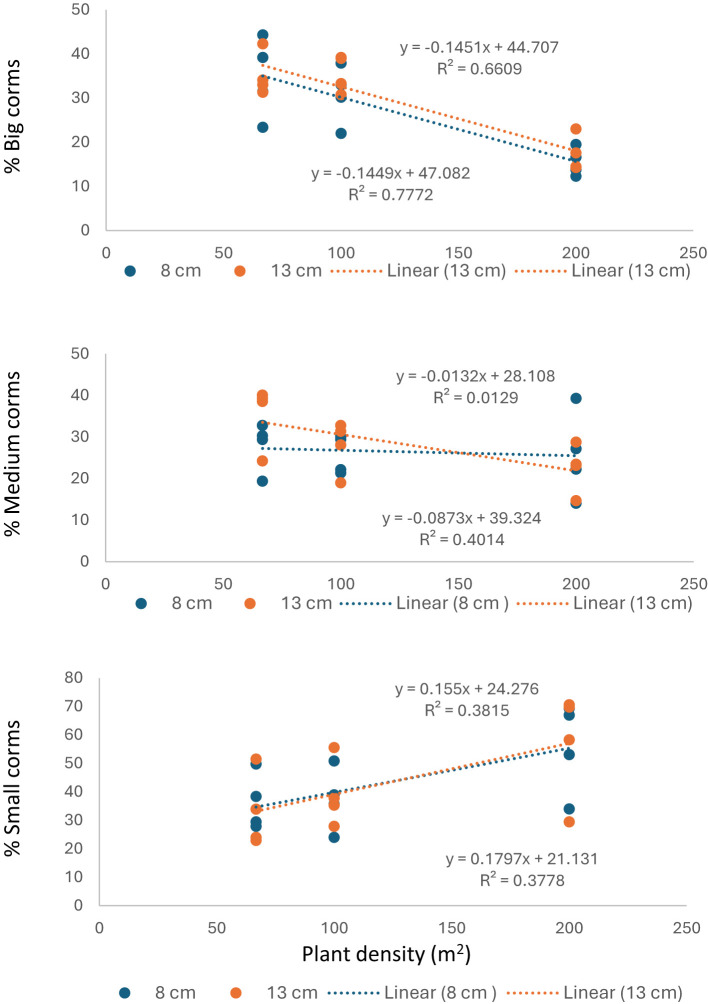
Relationship between plant density with percent of big, medium and small corms yield.

**Figure 8:**
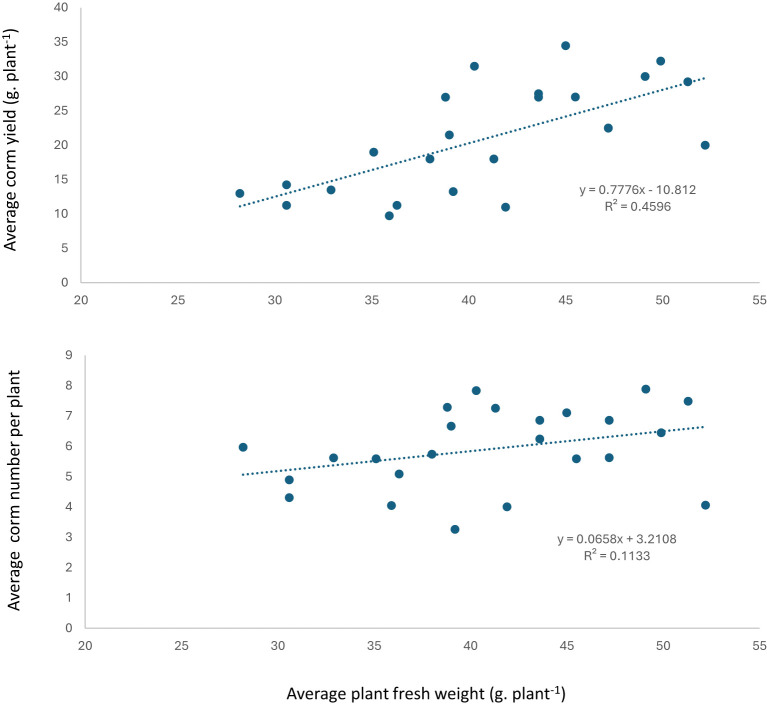
The relationship between plant fresh weight with corm yield and number of corms plant^-1^.

The relationship between planting density and new corms size showed that moderate to high (R^2^ = 0.66 and 0.77 for both planting depth) negative correlation between big size corms and planting density, while weak correlation with medium corms size except for 13cm planting depth and positive correlation with small corms size (**Figure 7**). Furthermore, positive correlation was observed between plant fresh weight and new corm weight yield, while positive weak correlation with number of new corms per plant (**Figure 8**).

## 4 Discussion

### 4.1 Climate

Saffron has been successfully grown in different geographic locations throughout the world. This crop can be cultivated in temperate, semi-arid, and arid climatic conditions (Kumar et al., 2009). However, saffron yield is highly affected by the corm properties and the conditions, and the environment corms are grown (Gresta et al., 2009; Baghalian et al., 2010; Siracusa et al., 2010). Rashed-Mohassel (2020) and Nehvi et al. (2010) reported that saffron sprouting, flower initiation, and time of flowering are the critical stages that are influenced by temperature and availability of water. Maximum day temperature of 23–25°C in September is essential for corm sprouting, whereas flowering is initiated when the maximum day temperature is below 17°C and a night temperature around 10°C (Gresta et al., 2009; Baghalian et al., 2010; Siracusa et al., 2010). Rashed-Mohassel (2020) stated a good correlation between air temperature and saffron yield. Lower temperatures in the period before flowering are associated with higher yields. Molina et al. (2005) reported that light and temperature are critical factors that affect saffron plant activities, including vegetative and generative growth, particularly the flowering habit.

In our trial, the maximum greenhouse temperature did not exceed 25°C in the first two months, showing that we achieved the recommended temperatures for optimum sprouting and flower initiation. However, temperatures during November and December were higher than the optimal for flower production and ranged between 17-20 °C, which accelerates vegetative growth and develops a large number of spathe but a smaller number of flowers. The maximum yield in our trial was 600 flowers m-2 and 5.6 g m-2 dry stigma, which was lower than expected, given that the big mother saffron corms we planted (about 25 g corm^-1^). This reduction in flower- and stigma yield might be due to these suboptimal air temperatures in this period. Pirasteh-Anosheh et al. (2023) reported that fields with higher yields were grown in lower temperatures. The lower temperatures induce the central corm buds, and root primordia appear at the base of corms (Negbi, 2006). Koocheki and Khajeh-Hosseini (2020); Negbi (2006); Pirasteh-Anosheh et al. (2022). Pirasteh-Anosheh et al. (2023) reported that autumn temperature is important for determining the yield of the current year, and spring temperature is important for determining the yield of the next year.

The maximum flower number (600 flowers m2) and dry stigma yield (5.6 g m2) were achieved by a plant density of 200 plants m2 and planting depth of 13 cm, followed by the same planting density and shallow depth (8cm) (552 flowers m^2^ and 5.2g m^2^, respectively). Many researchers study the effect of plant density and planting depth on stigma yield. Planting a high number of corms is expected to produce a high number of flowers when it is calculated per unit area (Yau and Nihmeah, 2004). However, when it is calculated per mother corm, the yield is reduced at high density due to high competition for light, water, and nutrient sources (El Hajj et al., 2019). Likewise, Behdani et al. (2008); Koocheki et al. (2016), Kumar et al. (2012), Nasseer et al. (2018), and Esmaeilian et al. (2022) reported that there was a positive correlation between flower yield and increasing plant density. Our results agreed with those reported by El Hajj et al., 2019), where a negative correlation was observed in average flower number per plant with increasing plant density (**Figure 1**). Reducing in planting density from 120 to 30 corms per m^2^, produced larger corms which improve yield potential and will result in greater saffron yield, and this compensates low planting density (Seyyedi et al., 2018). Planting 20 corms m^-2^ instead of 100 will reduce corm cost and maintain an acceptable yield over several years (Skinner et al., 2017). These data clearly show a trade-off between the yield for each corm and the yield per unit area. Hence, the optimum plant density will depend on economic factors, with on the one hand the costs of (imported) corms, production costs (greenhouse, cooling, labor), and the yield and price of saffron, and on the other, the yield and quality of produced mother- and baby corms.

Saffron propagation is done by the new corms (daughter corms) originating from the main corm (Sharaf-Eldin et al., 2013). Kaushal and Upadhyay (2002) reported that both the production of daughter corms and the yield of flowers depended on the mother corms’ size and environmental conditions (Rahimi et al., 2017). Temperatures between 10 and 20°C during January to Apr. favored plant vegetative growth and new corms production (Pirasteh-Anosheh et al., 2023). In our trial, the average day temperature during January to March ranged from 15 to 18°C inside the greenhouse, and these temperatures are more or less in agreement with the optimal temperatures for new corms development. However, in our trial, early leaf senescence, early March, likely affected new corms development. It is unknown what caused this early senescence; irrigation or plant nutrition are very likely causes, as fertigation continued the whole cropping period. Perhaps the high light intensity and high temperatures towards the end of this period could have caused this early dormancy.

Thus, our findings offer valuable guidance for optimizing saffron cultivation under greenhouse conditions, particularly in arid regions like Saudi Arabia, where extreme temperatures and water scarcity constrain open-field production. By demonstrating how specific planting depths and densities influence both flower yield and corm regeneration, this study provides a practical framework that growers in similarly hot, dry climates can adopt to enhance productivity and sustainability. The ability to achieve optimal sprouting and flower initiation by maintaining greenhouse temperatures below 25 °C during early growth, as shown in this trial, highlights the critical role of climate control in successful protected cultivation.

Where the cost and availability of quality corms are limiting factors, high planting densities may increase yield per unit area in the short term, but they compromise long-term sustainability due to reduced individual plant performance and limited propagation. Conversely, very low densities favor corm development but reduce overall yield efficiency. The recommended planting parameters offer a strategic balance—supporting both economic returns through viable stigma production and the regeneration of planting material through adequate daughter corm formation.

These findings lay the foundation for scalable saffron greenhouse systems in hot climates, providing a model that mitigates environmental stress while improving resource use efficiency. In this context, the study contributes not only to the agronomic optimization of saffron under controlled-environment agriculture but also to the adaptation of greenhouse practices to the specific climatic and economic conditions of Saudi Arabia.

While this study did not include measurements of secondary metabolites such as crocin, picrocrocin, and safranal—key indicators of saffron’s quality and commercial value—the existing literature suggests that these compounds are not directly influenced by the agronomic factors investigated here, namely planting density and depth, for instance, Koocheki and Seyyedi (2016) and Andabjadid et al. (2015) observed no significant variation in crocin or safranal content across different planting densities. Similarly, Sarwar et al. (2024) reported that although a planting depth of 15 cm enhanced corm emergence and vegetative vigor, it did not correlate with improvements in chemical quality traits. Instead, environmental factors such as temperature, precipitation, and soil moisture have been identified as more influential in determining secondary metabolite accumulation (Farrokhi et al., 2021; Gheshm and Brown, 2021). Recognizing this, future research should aim to integrate secondary metabolite profiling alongside agronomic assessments to provide a more comprehensive understanding of how both biophysical and environmental variables shape saffron quality across diverse cultivation settings.

## 5 Conclusion

Temperature, plant density, and planting depth strongly influence the development of flowers, the production of stigma, and the development of corms and daughter corms. Our results show that in a greenhouse with a controlled climate, the optimal temperature for saffron can be achieved, even in a hot climate, such as Saudi Arabia, where a temperature reduction of more than 15 degrees could be achieved inside the greenhouse. Further improvements are possible by achieving a lower temperature for the period after November. Our results confirm the data from the literature. In our trial, the highest production was at a density of 200 tubers m² and a planting depth of 13 cm, whereas the best corm size was found at the lowest density (67 corms m²). Ultimately, the optimal combination of planting density, planting depth, and temperature will depend on several factors. These include the cost of imported corms, production expenses, and labor. Greenhouse costs, energy use, and water for cooling will also influence production costs. On the other hand, saffron yield, quality, and the possibility of reusing corms and daughter corms for subsequent crop years will also play a key role in determining the best approach. The latest represents a critical area for further investigation in future research.

## Data Availability

The original contributions presented in the study are included in the article/supplementary material. Further inquiries can be directed to the corresponding author.
